# Abnormal static and dynamic functional connectivity of striatal subregions in patients with obsessive-compulsive disorder

**DOI:** 10.3389/fpsyt.2025.1529983

**Published:** 2025-02-21

**Authors:** Wenqing Shi, Ya Tian, Huiting Yang, Huirong Guo, Baohong Wen, Zijun Liu, Yong Zhang, Shaoqiang Han, Jingliang Cheng

**Affiliations:** ^1^ Department of Magnetic Resonance Imaging (MRI), The First Affiliated Hospital of Zhengzhou University, Zhengzhou, China; ^2^ Department of Psychiatry, The First Affiliated Hospital of Zhengzhou University, Zhengzhou, China

**Keywords:** obsessive-compulsive disorder, striatum, caudate, functional magnetic resonance imaging, dynamic functional connectivity

## Abstract

**Background:**

As a crucial node of the cortico-striato-thalamo-cortical (CSTC) loop, the striatum has long been considered to be involved in the pathophysiology of obsessive-compulsive disorder (OCD). Numerous neuroimaging studies have reported functional abnormalities of the striatum in OCD. However, altered dynamic functional connectivity (DFC) patterns of striatal subregions were rarely reported in patients with OCD.

**Methods:**

We collected resting-state functional MRI data from 97 first episode and drug-naïve OCD patients and 106 HCs matched for gender and age. Seed-based whole-brain resting-state functional connectivity (RSFC) and DFC analysis were performed for 12 striatal subregions. Between-group differences of the mean RSFC and DFC were determined using a two-sample t-test. In addition, we performed a Spearman’s correlation analysis to examine the relationship between altered RSFC and DFC and the clinical characteristics of OCD.

**Results:**

Patients with OCD exhibited increased RSFC between the superior ventral striatum (VSs) and the calcarine (CAL), lingual gyrus (LING), cuneus (CUN), supplementary motor area (SMA), precuneus (PCUN), paracentral lobule (PCL) and superior parietal gyrus (SPG). Increased RSFC between the left dorsal caudal putamen (DCP) and LING and inferior occipital gyrus (IOG) and increased RSFC between left ventral rostral putamen (VRP) and fusiform gyrus (FFG) were also found. in OCD group. The left dorsal caudate (DC) showed increased RSFC with CAL. In addition, OCD patients shows increased RSFC between multiple striatal seeds and cerebellum. The left VSs showed decreased DFC in the OCD patients with the PCUN, SPG and superior occipital gyrus (SOG). The right DC showed decreased DFC with the medial frontal gyrus orbital part (ORBmed), superior frontal gyrus orbital part (ORBsup) and gyrus rectus (REC). OCD severity was associated with DFC values between the right DC and ORBmed (r = 0.209, p = 0.044).

**Conclusion:**

Our study reveals disrupted RSFC and DFC between the striatal subregions and widespread brain regions in OCD patients. The findings highlight the role of the striatum in the neuropathology of OCD at a refined anatomical level and support the CSTC model in OCD.

## 1 Introduction

Obsessive compulsive disorder (OCD) is a common and disabling mental disorder characterized by obsessions and/or compulsions (1). Obsessions refer to intrusive and unwanted repetitive thoughts, impulses, images or urges. Compulsions are repetitive mental activities or behaviors that an individual feels driven to perform. These behaviors are typically stereotyped and ritualistic, such as repeatedly washing hands, checking locks, or counting objects. OCD typically causes significant anxiety or distress, affecting the individual’s daily life, social interactions, work, and studies. The course of OCD is typically chronic, spanning over years or decades, and is characterized by recurrent episodes (2, 3). OCD has a lifetime prevalence of 2–3% (4), causing significant negative impact on public health (5). However, the pathophysiology of OCD remains incompletely elucidated. Resting-state functional magnetic resonance imaging (R-fMRI) is a technique using the blood oxygenation level dependent (BOLD) signal to measure metabolic activity in brain region (6). Previous R-fMRI studies have reported abnormalities in functional connectivity (FC) in multiple brain regions in OCD, particularly in the striatum (7, 8).

As a crucial node of the cortico-striato-thalamo-cortical (CSTC) loop, the striatum has long been considered to be involved in the pathophysiology of obsessive-compulsive disorder. The striatum consists of the caudate nucleus and the putamen which receives synaptic input from entire cerebral cortex and integrates affective, motor, and cognitive information (9–11). Neuroimaging studies have suggested the striatum is involved in various motor-related function, executive/cognitive control and reward related/motivational processes (12–15). The striatum is a complex composed of structurally and functionally heterogeneous subregions. Nucleus accumbens, dorsal caudate and putamen separately constitute the affective circuit, dorsal cognitive circuit and ventral cognitive circuit (16). Previous studies have confirmed the functional segregation between the ventral and dorsal striatum in reward system, planning and executive functions, and learning processes (17–21). Di Martino et al. have subdivided striatum by defining six seed regions and using these six seeds to explore the whole-brain FC (22). Specifically, inferior ventral striatum (VSi) was functionally connected with regions involved in emotional processing while superior ventral striatum (VSs) was connected with regions involved in executive function, decision making, and motor planning. Dorsal caudate (DC) was primarily associated with the regions involved in cognitive control such as dorsolateral prefrontal cortex, ventral lateral prefrontal cortex, anterior cingulate cortex. Thus, the segmentation of striatum into subregions and using them as seed will significantly enhance the accuracy of whole-brain FC analysis, offering profound understanding into the intricate functional networks of the brain.

Previous research on resting-state fMRI in obsessive-compulsive disorder were under the premise that the brain remained static, failed to fully utilize the rich temporal dynamics inherent in spontaneous BOLD FC. In contrast to the static FC model, DFC assesses the time-varying covariance of neural signals across brain regions, enabling to explore the temporal characteristics of FC (23). The sliding window analysis method is the most common strategy for examining dynamic FC (24–26). DFC has been extensively applied to investigate the neuropathology of mental disorders including major depressive disorders (27–29), generalized anxiety disorders (30, 31), schizophrenia (32, 33) and epilepsy (34, 35). Ding et al. have found that the DFC between the left superior temporal gyrus and the left cerebellum Crus I and left thalamus, and between the right supplementary motor area and right dorsolateral prefrontal cortex and left precuneus was decreased in OCD (36). Teng et al.’s research reported that OCD exhibited significantly decreased DFC variability between the left thalamus seed and the left cuneus and right lingual gyrus as well as decreased DFC variability between the bilateral cuneus seed and bilateral postcentral gyrus (37). However, the DFC of the striatal subregions in patients with OCD remains unclear. In this research, we applied whole-brain voxel-based RSFC and DFC methods to explore the static and dynamic function alterations at rest in patients with OCD. Furthermore, we examined the association between RSFC and DFC alterations and the severity of OCD. In addition, previous studies have indicated that pharmacological treatment can altered the brain functional connectivity patterns in patients with OCD (38). We included first-onset OCD patients who had not previously received any medication.

## 2 Materials and method

### 2.1 Participants

Patients with OCD were recruited from out-patient/inpatient services of Department of Psychiatry, the First Affiliated Hospital of Zhengzhou University, Zhengzhou, China. Patients were diagnosed by a chief physician and a well-trained psychiatrist, following the guidelines outlined in the Diagnostic and Statistical Manual of Mental Disorders, Fifth Edition (DSM-V) for OCD. OCD patients were drug-naïve and should not have previous episodes. All patients were Han Chinese and right handedness. The severity of OCD was quantified using the Yale–Brown Obsessive Compulsive Scale (Y-BOCS) (39). The Self Rating Depression Scale (SDS) and the Self Rating Anxiety Scale (SAS) were used to evaluate depressive and anxiety symptoms. Furthermore, individuals who meet any of the following exclusion criteria will be excluded from participation in the current study: (1) Having comorbidity of other mental/psychotic disorders; (2) Taking drugs such as anesthesia and analgesia in the past 1 month; (3) History of alcohol or psychoactive substance abuse; (4) History of organic brain lesions, or other organic body disease; (5) Suffering from disorders of the nervous system, endocrine system, cardiovascular system, and respiratory system; (6) Contraindications for MRI scanning including implanted cardiac pacemakers, artificial heart valves, fixed dentures, metal braces, or other magnetizable foreign bodies in the body. Ninety-seven patients were included in the case group after meeting the above inclusion criteria. We recruited 106 HCs matched for gender and age from the general public through poster advertisement. The inclusion criteria for the HC group are: (1) Han Chinese and right-handed; (2) No obvious abnormalities were found in the head MRI; (3) No mental disorders or neurological diseases; In addition, exclusion criteria for HC group include the following: (1) Suffering from severe systemic diseases; (2) Alcoholism or substance abuse; (3) family history of hereditary neurological disorders; (3) Claustrophobia or having any metallic objects in the body.

All participants signed written informed consent forms before experiment. The study received approval from the research ethical committee of the First Affiliated Hospital of Zhengzhou University.

### 2.2 Data acquisition

The R-fMRI data of participants were acquired using on 3-Tesla GE Discovery MR750 scanner (General Electric, Fairfield Connecticut, USA). During R-fMRI scanning, all subjects were instructed to keep their eyes closed but stay awake, and avoid consciously engaging in thoughts or memories. First, each subject underwent conventional imaging sequences including T1-weighted, T2-weighted, and T2-weighted fluid-attenuated inversion recovery (FLAIR) to exclude organic lesions or other abnormalities. The scanning parameters employed in the resting-state functional scans were as follows: repetition time = 2000ms; echo time = 30ms; number of slices = 32; thickness = 4 mm; resolution matrix = 64 × 64; flip angle = 90°; field of view= 220 × 220 mm2; and slice gap = 0.5mm.

### 2.3 Data processing

The Data Processing & Analysis for Brain Imaging toolbox (DPABI, version 4.3; http://rfmri.org/dpabi) (40) was used for the analysis of R-fMRI data within a pipeline framework. The main steps were as follows: (1) removing the first 10 time points; (2) slice-timing correction and realignment; (3) correction of head motion (excluding data with translation over 3 mm or rotation over 3 degree); (4) spatial normalization into the standard Montreal Neurological Institute template (resampling voxel size, 3 × 3 × 3 mm3); (5) spatial smoothing with a Gaussian kernel of full-width at half-maximum of 6 mm; (6) detrending of BOLD signals for reducing low-frequency drift; (7) temporal band pass (0.01 and 0.1). Four subjects in the OCD group and two subjects in the HC group were excluded because head motion exceeded the previously set threshold. The final sample included 93 OCD patients and 104 HCs.

### 2.4 Dynamic and static FC analyses

Based on the research by Di Martino and his colleagues, we used 12 seeds of striatum for whole-brain RSFC and DFC. For each hemisphere, six MNI coordinates were designated and seeds were defined as spherical mask with a 4mm radius centered around each coordinate. (22) (see **Table 1**). DPARSF was used for RSFC analysis. The mean time series of activity within this seed region was computed. Then, Pearson correlation analysis was used to calculate the temporal correlation between the mean time series of seed region and the time series of each voxel across the whole brain. The correlation coefficients were then transformed into Z-scores using Fisher’s r-to-z transformation to improve the normality of the data. Dynamic FC analysis was performed using Dynamic Brain Connectome (DynamicBC, version 2.0; http://restfmri.net/forum/DynamicBC) analysis toolbox (41). Sliding time-window analysis was adopted to characterize FC temporal dynamics. The window length is a crucial parameter, but there is currently no consensus on its selection. Based on previous research, the minimum window length should be at least 1/*fmin* to prevent the introduction of spurious fluctuations caused by shorter intervals. Here,*fmin* is the minimum frequency of time series (42). Simultaneously, the window length should not be excessively long to prevent disruption of the temporal variability dynamics of FC. (43). We set the length of the window to 50TR (1TR=2s) with an overlap of 0.8 which means the step size is 10TR. Within each window, we applied seed-based DFC analysis to compute the temporal correlation coefficients between the averaged time course of each ROI and all other voxels to build a FC map. Then, a Fisher’s r-to­z transformation was applied for the resulting FC matrices in order to improve the normality of correlation distribution. For each subject, the standard deviation of zFC values was calculated to obtain the DFC values of all voxels within windows.

**Table 1:** Coordinates for right and left hemisphere seeds defined in the MNI stereotaxic space.

ROI	x	y	z
VSi	9	9	-8
VSs	10	15	0
DC	13	15	9
DCP	28	1	3
DRP	25	8	6
VRP	20	12	-3

ROI, regions of interest; VSi, inferior ventral striatum; VSs, superior ventral striatum; DC, dorsal caudate; DCP, dorsal caudal putamen; DRP, dorsal rostral putamen; VRP, ventral rostral putamen.

### 2.5 Statistical analyses

The statistical analysis was conducted using the statistical SPSS software (IBM SPSS Statistics for Windows, version 26.0. Armonk, NY: IBM Corp). Demographic and clinical data were analyzed using the chi-squared test for gender and independent two-sample t-tests for the other demographic characteristics between the two groups. The RSFC and DFC values were compared between the OCD group and HCs using voxel-wise two-sample t-tests in SPM12 toolbox, with age, gender, education level, and head motion included as covariates. The results were thresholded using Gaussian random field (GRF) correction, carried out via the DPABI toolbox, with a voxel-level threshold of p < 0.005 and a cluster-level threshold of p < 0.05. To examine the association of RSFC and DFC values with disease severity, we extracted the mean RSFC and DFC values from brain areas showing significant between-group differences and performed a correlation analysis with Y-BOCS total scores, SDS and SAS scores. Spearman’s correlation was analyzed through SPSS with Bonferroni correction.

### 2.6 Validation analyses

To verify the reliability of our findings on DFC, we used two additional window lengths of 60TR and 80TR both shifted by 10 TR to eliminate the impact of parameter selection and confirm the stability of DFC.

## 3 Results

### 3.1 Demographic and clinical data of patients

The clinical demographics were presented in **Table 2**. 93 OCD patients and 104 HCs were included in RSFC and DFC analyses. No significant group differences appeared in terms of sex composition, and age (p > 0.05). The HC group had significantly higher educational levels than the OCD group (p<0.05). The Y-BOCS scores of the OCD patients were higher than those of the HC group.

**Table 2:** Demographic and clinical characteristics of participants.

	HC (N = 104)	OCD (N= 93)	p
Gender(male/female)	51/53	44/49	0.809
Age, mean (SD) [range], y	23.14 (5.64) [16-43]	23.31 (9.45) [12-49]	0.882
Educational level, mean (SD), y	15.21 (3.17)	11.88 (3.00)	<0.05
Y-BOCS scores, mean (SD), [range]	–	22.57 (5.99) [10-40]	
Anxiety		25.22 (12.49)	
Depression		36.68 (13.52)	
Handedness, right/left	104/0	93/0	

OCD, obsessive-compulsive disorder; HC, healthy control; Y-BOCS, the Yale-Brown Obsessive Compulsive Scale; SD, standard deviation.

### 3.2 The resting-state functional connectivity analyses

**Figure 1:**
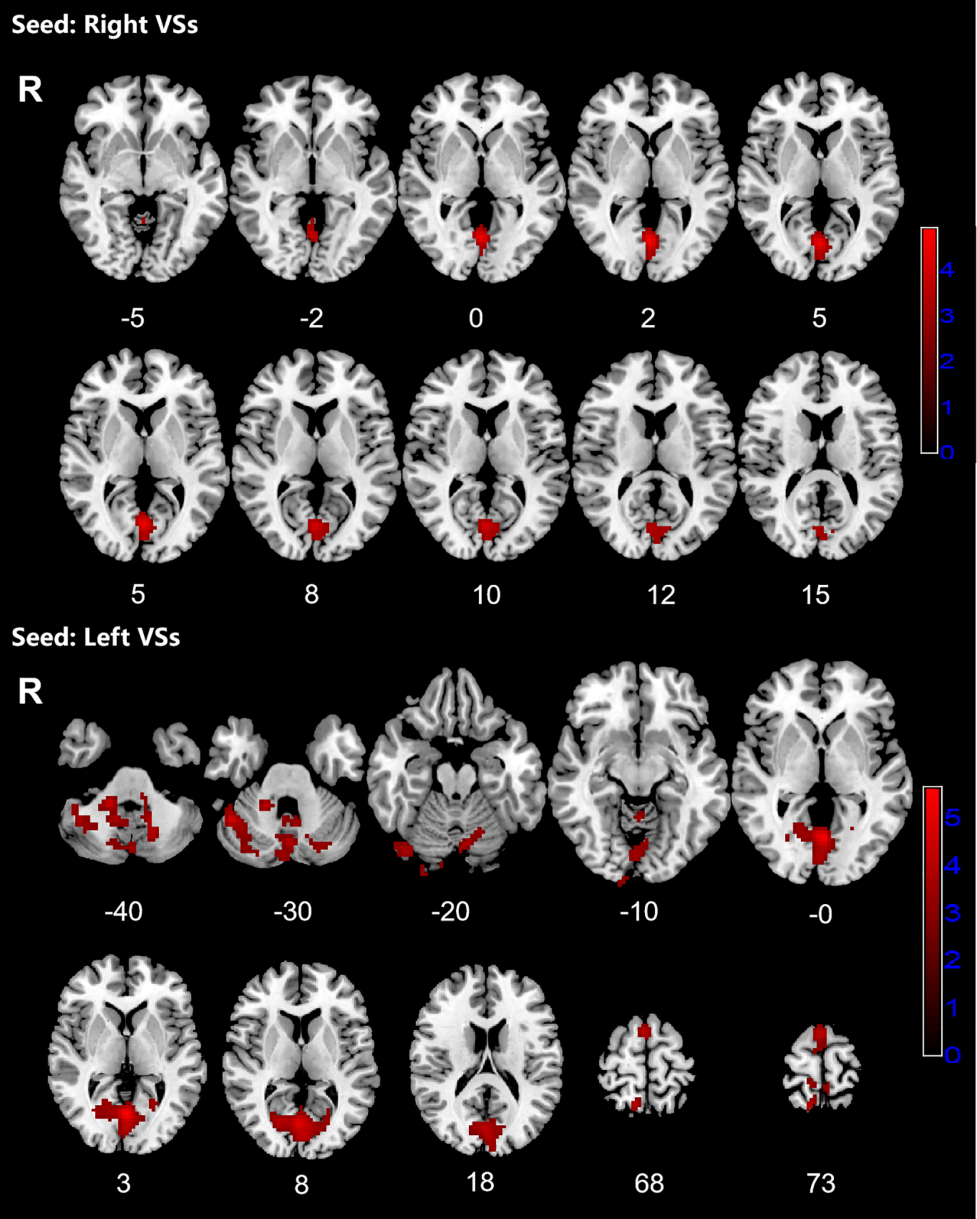
Significant increased resting-state functional connectivity (RSFC) of the bilateral superior ventral striatum (VSs) in OCD patients, compared with HC. The red regions in the brain slices present the location of difference (GRF corrected).

**Figure 2:**
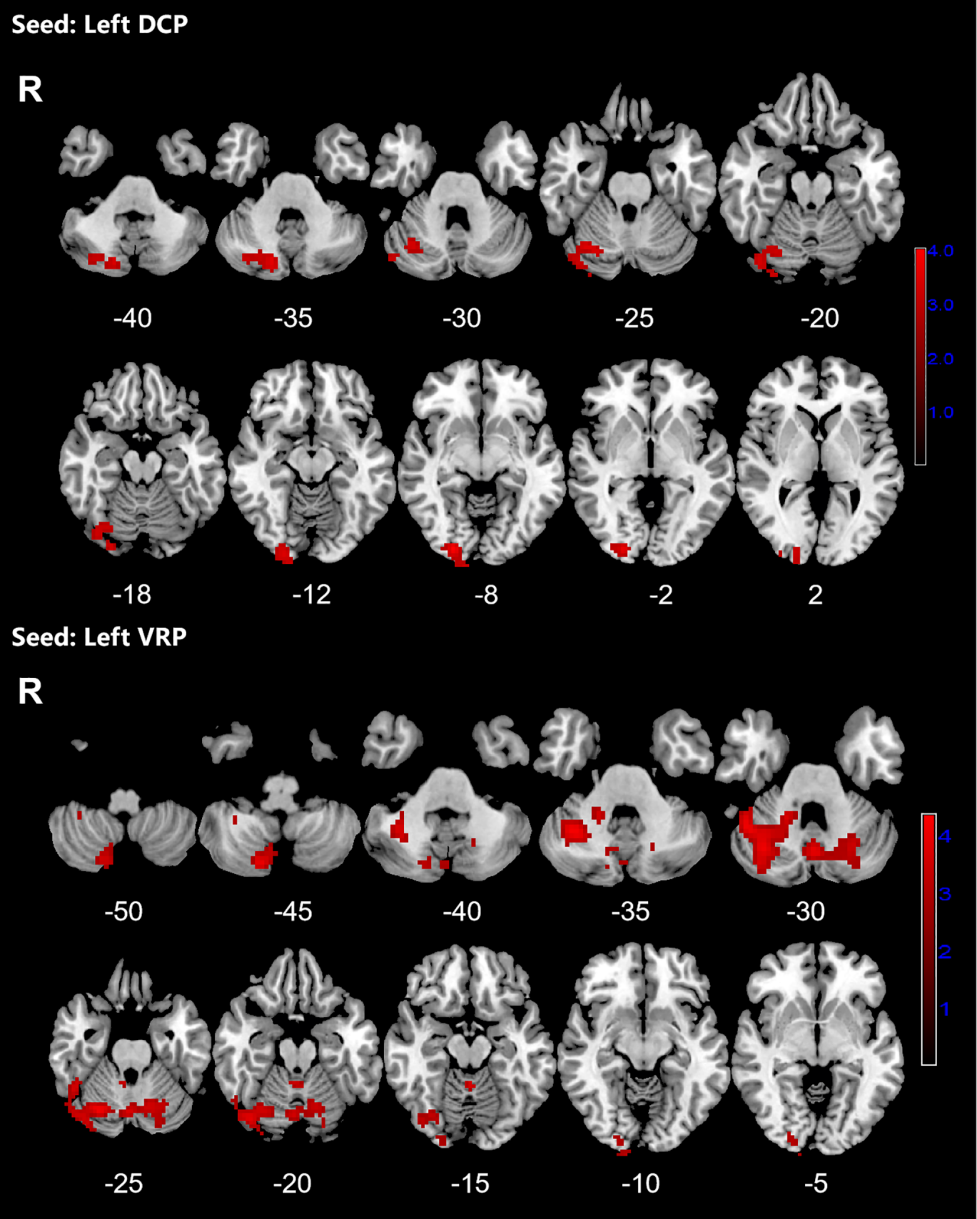
Significant increased resting-state functional connectivity (RSFC) of the left dorsal caudal putamen (DCP) and ventral rostral putamen (VRP) in OCD patients, compared with HC. The red regions in the brain slices present the location of difference (GRF corrected).

**Figure 3:**
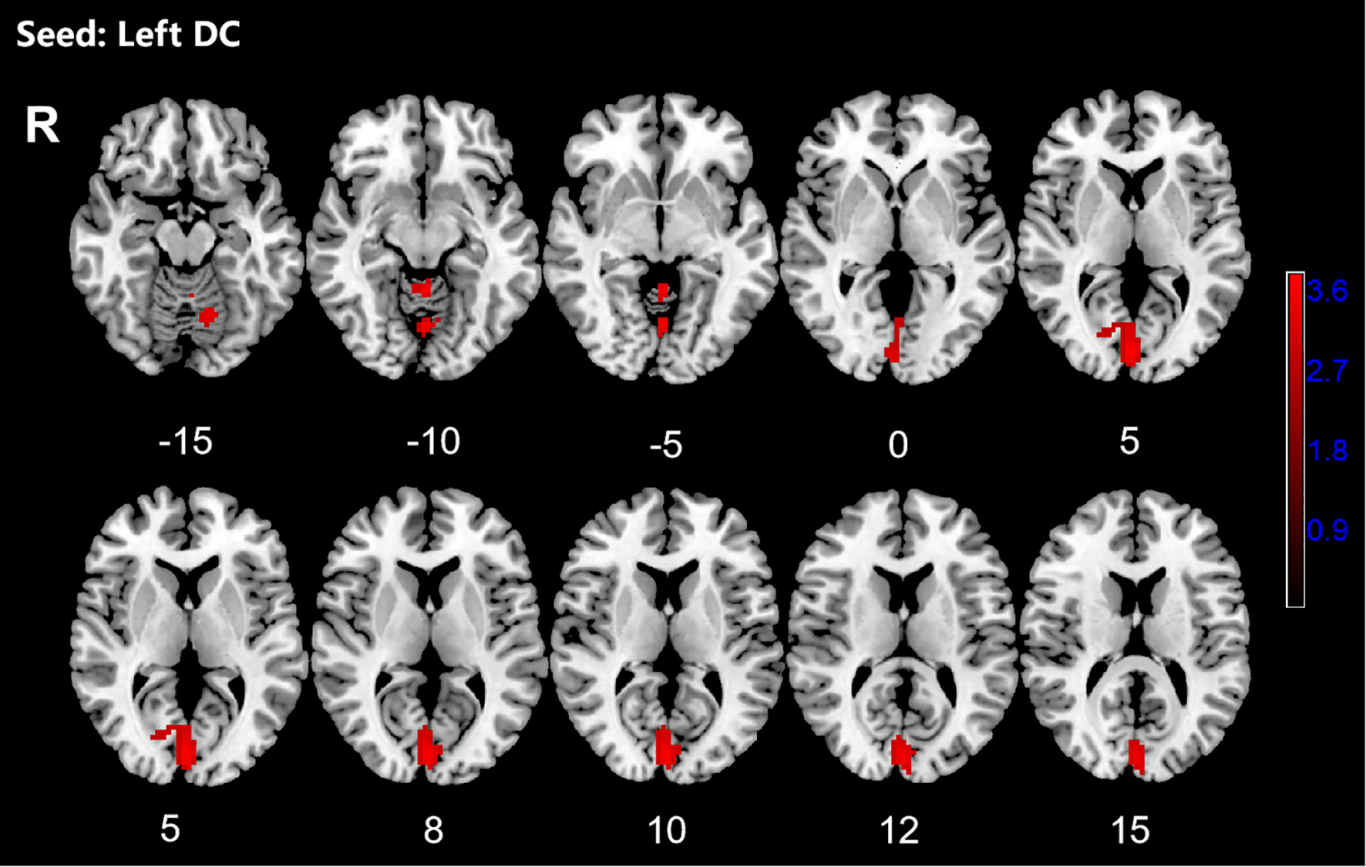
Significant increased resting-state functional connectivity (RSFC) of the left dorsal caudate (DC) and in OCD patients, compared with HC. The red regions in the brain slices present the location of difference (GRF corrected).

Patients with OCD exhibited increased RSFC between the right VSs and bilateral CAL and LING and increased RSFC between the left VSs and bilateral cerebellum, CAL, CUN, LING, SMA, PCUN, PCL and right SPG. Increased RSFC between the left DCP and right cerebellum, right LING and right IOG and increased RSFC between the left VRP and bilateral cerebellum and right FFG in OCD group were also found. Besides, the left DC showed increased RSFC with bilateral CAL and left cerebellum (**Figures 1**–**3**; **Table 3**).

**Table 3:** Brain regions showed increased RSFC with striatal subregions.

Seeds	Clusters	Voxels	Brain regions(L/R)	Peak MNI (x,y,z)	T-value
Right VSs	1	171	calcarine (L/R)	0, -69, 3	4.8481
			lingual gyrus (L/R)		
Left VSs	1	2101	cerebellum lobuleVIII (L/R)	0, -69, 3	5.5771
			calcarine (L/R)		
			cerebellum CrusI (L/R)		
			cuneus (L/R)		
			lingual gyrus (L/R)		
			cerebellum lobuleVI (L)		
			cerebellum lobuleIX (L/R)		
			cerebellum CrusII (R)		
			Vermis IV-V		
	2	417	supplementary motor area (L/R)	18 -51 57	4.2684
			precuneus (L/R)		
			superior parietal gyrus (R)		
			paracentral lobule (L/R)		
Left DC	1	247	calcarine (L/R)	-3, -48, -9	3.7503
			cerebellum lobuleVI (L)		
Left DCP	1	347	cerebellum CrusI (R)	24, -90, -6	4.016
			lingual gyrus (R)		
			inferior occipital gyrus (R)		
			cerebellum CrusII (R)		
			cerebellum lobuleVI (R)		
Left VRP	1	323	cerebellum lobuleVI (L)	12, -75, -48	4.0939
			cerebellum CrusI (L)		
			Cerebellum lobuleVIII (R)		
	2	555	cerebellum CrusI (R)	39, -54, -33	4.3466
			cerebellum lobuleVI (R)		
			fusiform gyrus (R)		

MNI, Montreal Neurological Institute; VSs, superior ventral striatum; DC, dorsal caudate; DCP, dorsal rostral putamen; VRP, ventral rostral putamen; L, left; R, right; +, positive; –, negative.

### 3.3 Dynamic functional connectivity analyses

**Figure 4:**
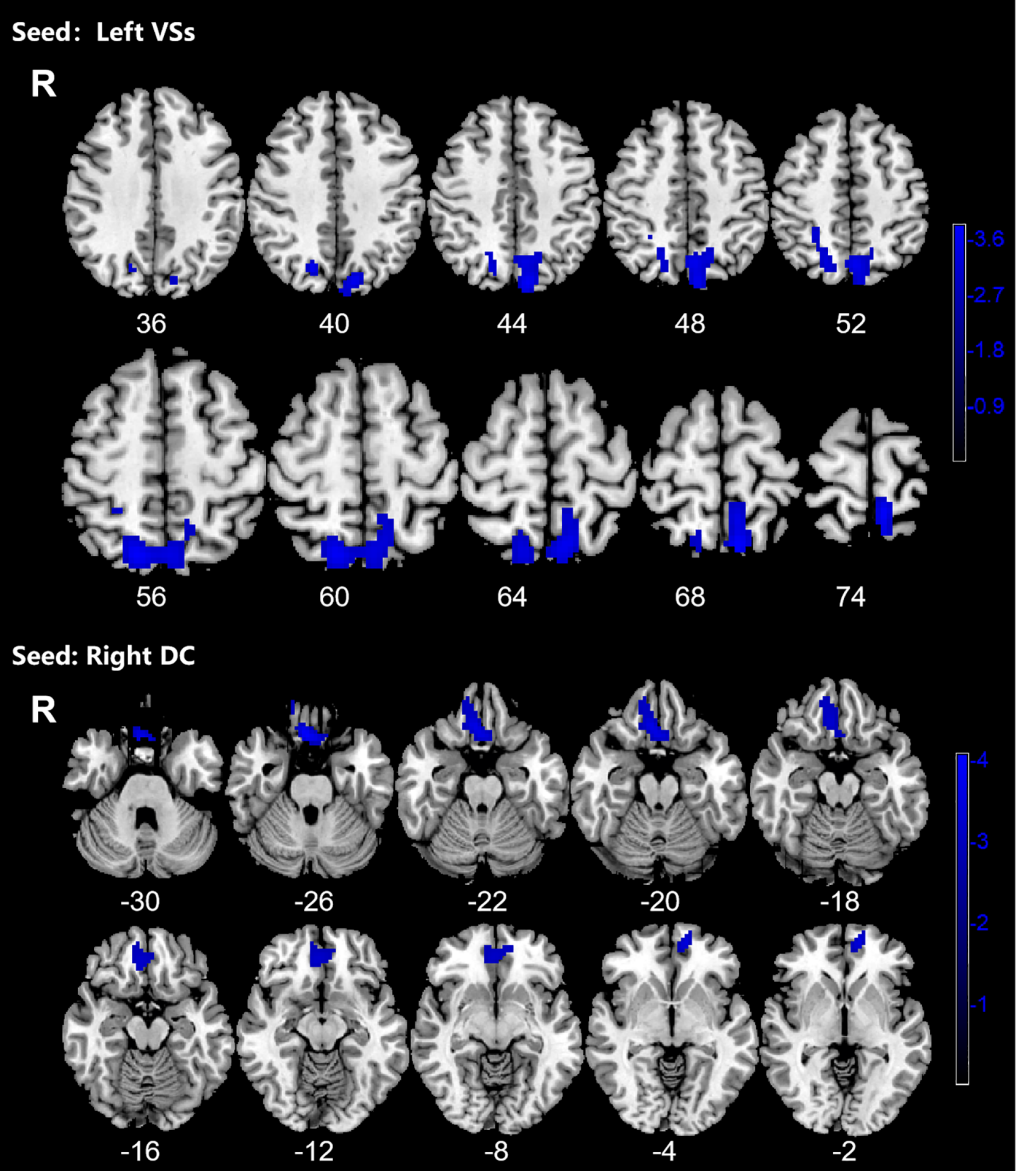
Significant decreased dynamic functional connectivity (DFC) of the left superior ventral striatum (VSs) and dorsal caudate (DC) in OCD patients, compared with HC. The blue regions in the brain slices present the location of difference (GRF corrected).

OCD group displayed significantly decreased DFC between the left VSs and bilateral PCUN, SPG and left SOG and decreased DFC between the right DC and bilateral ORBmed, REC and right ORBsup (**Figure 4**; **Table 4**).

**Table 4:** Brain regions showed decreased DFC with striatal subregion.

Seeds	Clusters	Voxels	Brain regions (L/R)	Peak MNI (x,y,z)	T-value
Left VSs	1	625	precuneus(L/R)	-9, -63, 69	-3.7987
			superior parietal gyrus(L/R)		
			superior occipital gyrus (L)		
Right DC	1	319	gyrus rectus (L/R)	3, 21, -24	-4.0634
			medial frontal gyrus, orbital part (L/R)		
			superior frontal gyrus, orbital part (R)		

MNI, Montreal Neurological Institute; VSs, superior ventral striatum; DC, dorsal caudate; L, left; R, right; +, positive; –, negative.

### 3.4 Spearman correlation analyses

**Figure 5:**
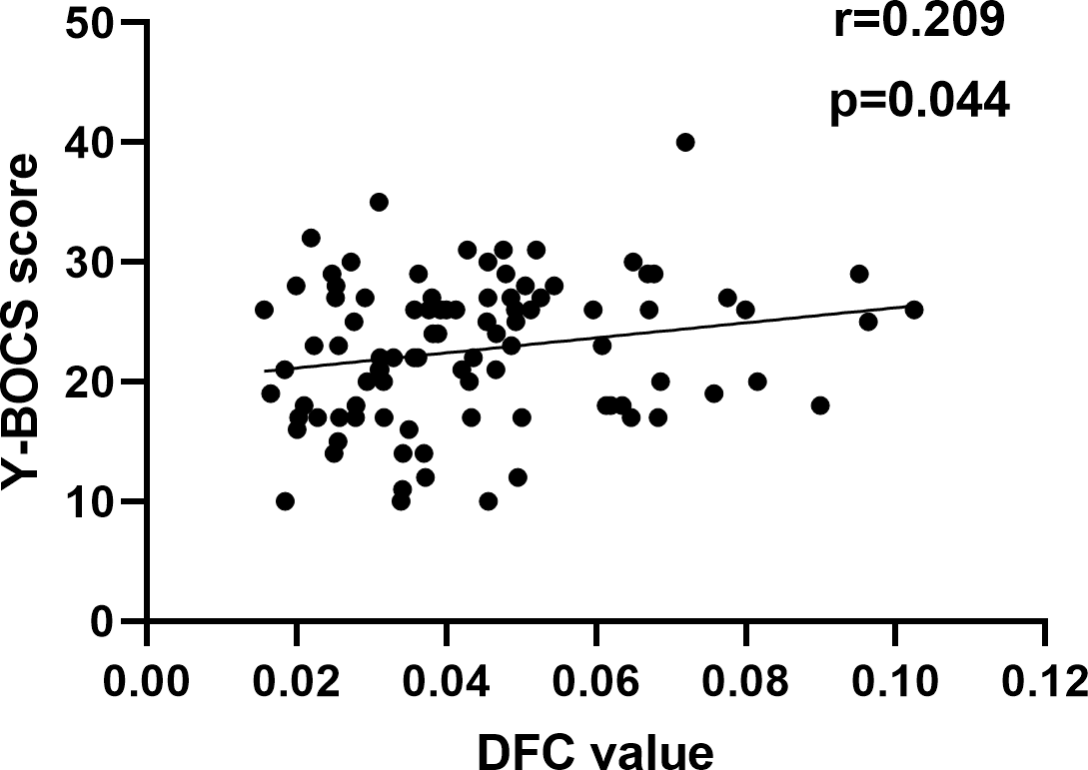
Correlation between altered DFC and Y-BOCS. A positive correlation between the altered DFC of the left ORBmed and Y-BOCS scores.

Since the data for correlation analyses does not follow a normal distribution, we conduct a Spearman correlation analysis. The Spearman correlation analysis showed that decreased DFC of the left ORBmed was significantly related to Y-BOCS scores in OCD (**Figure 5**). However, the significance did not survive the Bonferroni correction (p < 0.05/10 = 0.005). The correlation analysis of brain regions with Y-BOCS scores are provided in **Supplementary Table S3** in the **Supplementary Materials**. We did not find the correlation between the increased RSFC and Y-BOCS total scores. In addition, we did not find the correlation between the altered RSFC and DFC and anxiety and depression symptoms.

### 3.4 Validation results

The results of DFC between two groups using sliding window lengths of 60TR and 80TR were similar to those of DFC with sliding window length = 50 TR. The results of DFC with a window length of 60TR were showed in **Supplementary Figure S1**, and **Supplementary Table S1** and the results of DFC with a window length of 80TR were showed in **Supplementary Figure S2**, and **Supplementary Table S2** (see in the **Supplementary Material**).

## 4 Discussion

The present study examined the whole-brain RSFC and DFC using the subregions of the striatum as seeds in first-episode, drug-naïve OCD patients compared to healthy controls. We found that in OCD patients, the increased RSFC were mainly located within the cerebellum, SMA, parietal and occipital regions including CAL/LING, CUN, PCUN, PCL SPG, IOG. The results showed decreased DFC values between the left VSs and PCUN, SPG and SOG and decreased DFC values between the right DC and the ORBmed, ORBsup and REC. The correlation analysis showed that decreased DFC values of the ORBmed was significantly related to the severity of OCD.

In this study, we observed multiple striatal seeds such as bilateral VSs, left DC, left DCP showed increased RSFC with CAL and LING in patients with OCD. The CAL and LING are located in the occipital lobe and are parts of the visual cortex. The CAL plays an important role in integrating “visuopsychic” and “visuosensory” processing (44). The lingual gyrus is involved in the processing of visual information (45–47). Evidence from previous study suggests that abnormalities of visual perception involve in the neuromechanisms of OCD (48). A previous fMRI study showed that the lingual gyrus was activated when viewing neutral faces relative to scrambled images (49). The increased RSFC between the striatum and CAL and LING and may lead to overreaction to certain visual stimuli, triggering intense, involuntary and repetitive thoughts and behaviors.

We found the left VSs showed increased RSFC with the SMA, SPG and PCL. The SMA is located on the medial surface of the frontal lobe and plays an important role in movement planning and execution, as well as cognitive control (50–52). The SMA is part of the ‘sensorimotor’ CSTC of OCD, involving sensorimotor processes (1). A previous study foud that OCD patients exhibited excessive activation in the SMA during error processing (53). Another study showed increased RSFC between the right pre-SMA and the inferior frontal gyrus (IFG), and the increased FC was associated with impaired response inhibition in OCD patients (54). The SMA is also a key target for repetitive transcranial magnetic stimulation (rTMS) in the treatment of OCD. Multiple studies have shown that rTMS targeted at the SMA can effectively alleviate OCD symptoms (55–57). The PCL and SPG are also considered an important motor area, possibly related to the mental representation of movement (58). The increased functional connectivity between VSs and SMA, SPG and PCL may reflect deficits in cognitive and motor control.

Our results showed increased RSFC between multiple striatal seeds and the cerebellum. The cerebellum is traditionally thought to be involved in motor coordination and balance. Increasing evidence suggests that the cerebellum plays an important role in cognitive and emotional processes (59–61). The involvement of cerebellum in the pathophysiology of OCD has been confirmed. Previous studies have reported that OCD patient showed altered amplitude of low frequency fluctuations (fALFF) in cerebellum (62–64). Nabeyama et al. found that after behavioral therapy, OCD patients showed increased activation of the cerebellum during cognitive tasks (65). Abnormal functional connectivity between the cerebellum and widespread brain networks has been reported (66, 67). One previous study found increased global brain connectivity (GBC) in the putamen and cerebellar cortex of OCD patients (68). Zhang et al. found weakened functional connectivity between the cerebellar regions and the striatum (63). Differing from their findings, we observed increased RSFC between multiple striatal subregions and cerebellar regions. This could be attributed to functional heterogeneity of the striatum. Researches indicates that the basal ganglia and cerebellum are interconnected via dense disynaptic pathways, influencing cortical input and output (69–71). The deep cerebellar nuclei (DCN) neurons have a significant impact on striatal neuron activity through their output pathways, thereby modulating behavior related to reward processing (72). The enhanced FC between the striatum and cerebellum may be associated with impairments in executive control and the reward system.

It is worth noting that we found increased RSFC and decreased DFC between the left VSs and the precuneus in OCD patients compared with the HCs. Precuneus is part of the default mode network, involving in self-consciousness and self-referential processing (73–75). It plays a crucial role in a diverse array of cognitive functions, including visuo-spatial imagery, episodic memory retrieval and self-processing operations (76). It has been found that patients with OCD exhibit excessive activation of the precuneus when performing higher-order cognitive tasks, such as repetitive visual stimuli, working memory-related visuospatial task, or obsessive–compulsive symptom provoking tasks (77–81). Neuroimaging studies of resting-state functional imaging have reported abnormal functional connectivity between precuneus and widespread brain areas including parietal lobe, sensorimotor area, visual cortex, cerebellum, angular gyrus, and middle frontal gyrus (82, 83). A previous study reported OCD patients showed decreased functional connectivity between the caudate nucleus and precuneus during psychological distress, which was positively correlated with the severity of compulsive symptoms (84). The enhanced RSFC between the VSs and the precuneus may reflect an over-synchronization of these two regions in cognitive control and emotional regulation. As higher DFC indicates superior information processing capability and enables higher cognitive and behavioral flexibility (85). The reduced DFC between the left VSs may account for impairment of cognitive functions, self-referential processing in patients with OCD.

We also found the decreased DFC value between the right DC and the bilateral ORBmed, REC and right ORBsup. The orbitofronto-striatal circuit has consistently been thought to be associated with the neuropathology of OCD (86). Evidence from previous research indicates that the orbital frontal cortex (OFC) plays an important role in emotional regulation, reward processing, and decision-making (87–92). Functional brain imaging studies have demonstrated significantly increased OFC activity at rest in OCD (93, 94). A series of paradigm studies have reported abnormal activation of the OFC in OCD patients such as symptom provocation task, reversal learning and go/no-go task (95–98). Previous study reported increased functional connectivity between the striatal subregions and the OFC in OCD patients. (7, 99). The OFC plays an important integrative role between the internal state (regulated by the hypothalamus) and the external state (regulated by the striatum) (100). It is reported DC is more specifically associated with working memory and executive function. (16). The decreased DFC between DC and OFC may indicate reduced efficiency in information transfer between these brain regions, leading to the dysfunction of reward and executive system. In addition, we observed the DFC values of OFC correlate positively with YBOCS scores, suggesting that dysfunctions in OFC play a substantial role in OCD symptomatology. Nakao et al. found decreased activation in the OFC related to symptom provocation after symptom improvement (96). Researches have shown that OFC activity decreased with treatment response but increased with symptom provocation. Additionally, some studies reported that after treatment, there is a decrease in caudate glucose metabolism, while the caudate nucleus is activated during symptom provocation (101). As the severity of OCD increases, the enhanced DFC may reflect compensation for the excessive activity of the OFC, aiming to strengthen regulatory capacity by increasing interactions with other brain regions.

There remain a few possible limitations to consider in this study. First, there is no agreement on the selection of window length for the sliding-window method and different window lengths may lead to varied results. A shorter window may lead to higher noise and variability, while a longer window may fail to capture rapid dynamic changes (42, 43). We choose a window length of 50TR based on a comprehensive consideration of the data characteristics and experimental design. We conducted validation analysis that confirmed the stability of our results. Second, the correlation analysis did not show statistical significance after applying the Bonferroni correction. The lack of significance in the results may be attributed to factors such as a small sample size, weak correlations between variables, and overly stringent Bonferroni correction. It is important to note that even in the absence of statistical significance, the observed trends are still clinically significant and provide insights for future studies. In the future, more patients need to be included or other statistical correction methods can be used. Third, we did not find abnormal DFC using the putamen seed, contrary to many previous studies that have reported aberrant function of putamen in OCD. Therefore, it is important to emphasize that this is an exploratory study before drawing any conclusions.

## 5 Conclusion

This study explored the RSFC and DFC between striatal subregions and whole-brain areas in patients with OCD. We found increased RSFC and decreased DFC between the striatal subregions and widespread brain regions. Our findings expand on the existing literature and support the critical role of the striatum in the neuropathology of OCD.

## Data Availability

The datasets presented in this article are not readily available because the dataset is for internal use only by the Magnetic Resonance Imaging Department of the First Affiliated Hospital of Zhengzhou University. Requests to access the datasets should be directed to JC.
