# Supplementary material for: Abnormal static and dynamic functional connectivity of striatal subregions in patients with obsessive-compulsive disorder
Source: Front Psychiatry. 2025 Feb 21;16:1529983. doi: 10.3389/fpsyt.2025.1529983 (PMC11885261; doi:10.3389/fpsyt.2025.1529983)
Supplement: Supplementary file 1 [file Supplementaryfile1.pdf]

Supplementary Material

FigureS1

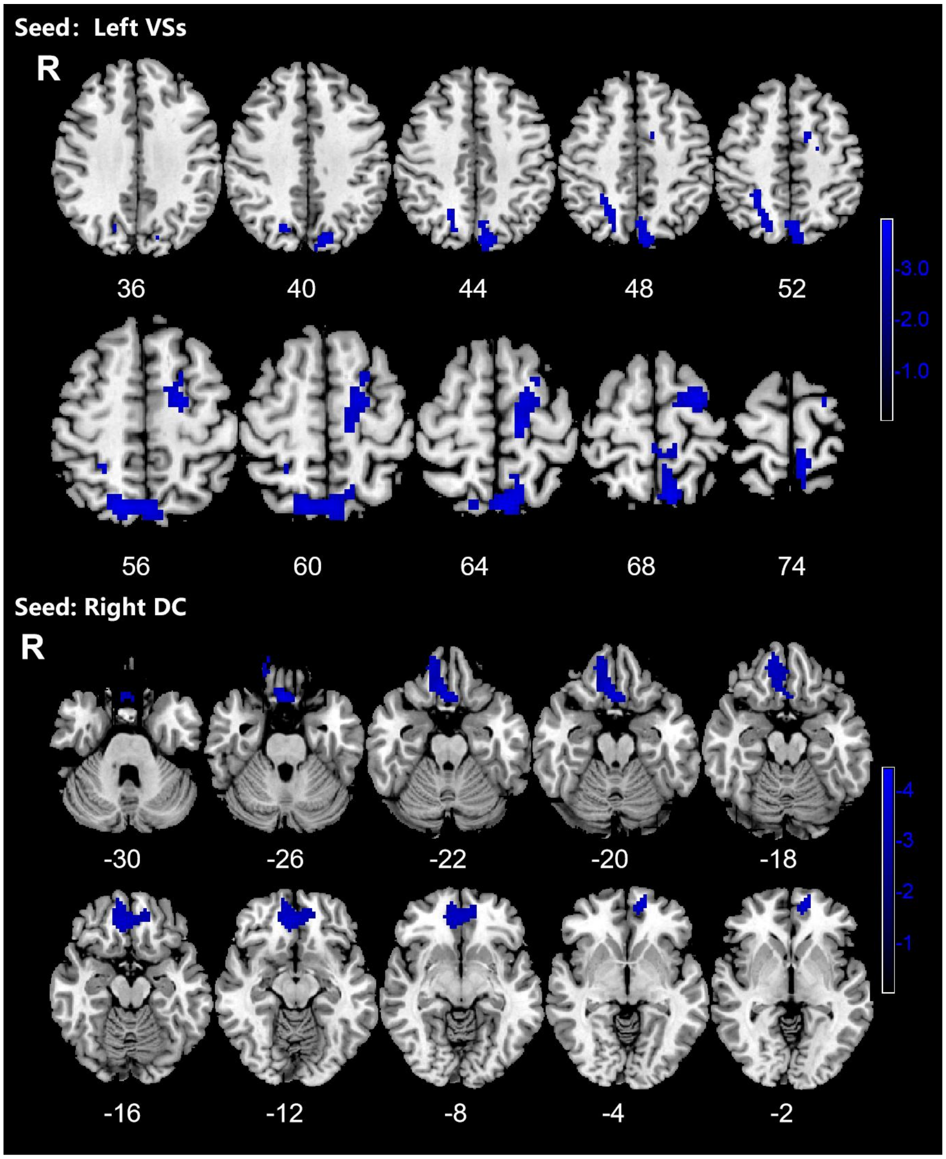

Note: Significantly decreased dynamic functional connectivity (DFC) of the left superior ventral striatum (VSs) and the right dorsal caudate (DC) in patients, compared with HC. The blue regions in the brain slices present the location of difference (GRF corrected).

Figure S2

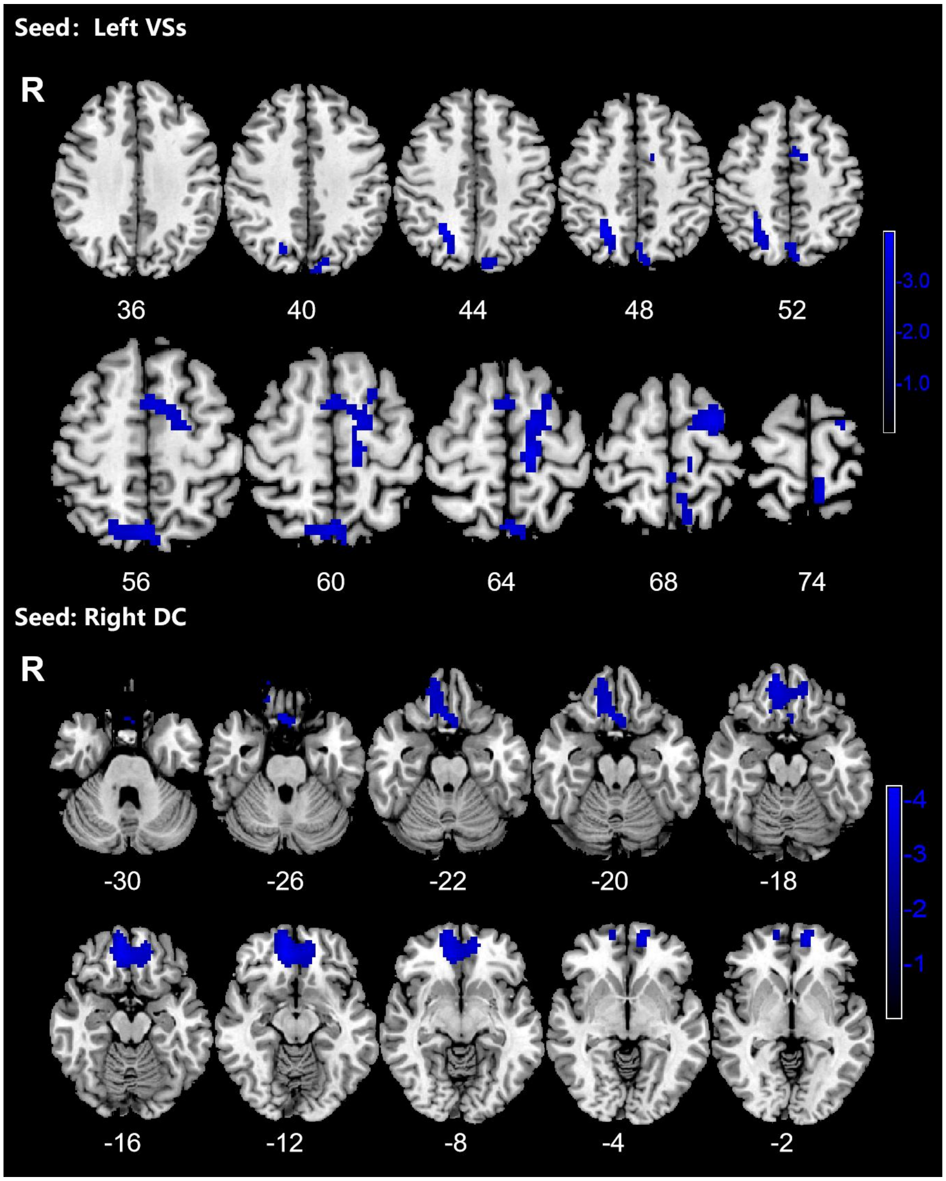

Note: Significantly decreased dynamic functional connectivity (DFC) of the left superior ventral striatum (VSs) and right dorsal caudate (DC) in patients, compared with HC. The blue regions in the brain slices present the location of difference (GRF corrected).

TableS1 Brain regions showed decreased DFC with striatal subregions

Seeds	Clusters	Voxels	Brain regions(L/R)	Peak MNI (x,y,z)	T
Left VSs	1	635	precuneus (L/R)	-24, -6,72	-3.9296

			superior frontal gyrus (L/R)		
			Left paracentral lobule		
			superior occipital gyrus (L)		
			supplementary motor area (L)		
			superior parietal gyrus (L)		
Right DC	1	403	gyrus rectus (L/R)	3, 21, -24	-4.39
			medial frontal gyrus, orbital part (L/R)		
			superior frontal gyrus, orbital part (R)		
			superior frontal gyrus, medial (L)		

MNI, Montreal Neurological Institute; VSs, superior ventral striatum; DC, dorsa caudate; L, left; R, right; +, positive; –, negative

TableS2 Brain regions showed decreased DFC with striatal subregions

Seeds	Clusters	Voxles	Brain regions (L/R)	Peak MNI (x,y,z)	T
Left VSs	1	297	precuneus (L/R)	24, -57,48	-3.9658
			superior parietal gyrus (R)		
			superior occipital gyrus (L/R)		
	2	256	superior frontal gyrus (L)	-27, -3,72	-3.9771

			supplementary motor area (L)		
			paracentral lobule (L)		
Right DC	1	523	medial frontal gyrus, orbital part (L/R)	-12,60,0	-4.1832
			gyrus rectus (L/R)		
			superior frontal gyrus, orbital part (L/R)		
			superior frontal gyrus, medial (L)		

MNI, Montreal Neurological Institute; VSs, superior ventral striatum; DC, dorsa caudate; L, left; R, right; +, positive; –, negative

TableS3 Results of correlation analysis

Brain regions	Peak MNI (x,y,z)	Number of voxels	T value	Y-BOCS (r, p)
Left precuneus	-9, -63, 69	287	-3.7987	-0.051, 0.611
Right superior parietal gyrus	5, -68, 56	89	-3.7227	-0.032, 0.758
Right precuneus	12, -68,57	83	-3.6368	-0.073, 0.489
Left superior occipital gyrus	-10, -81,45	48	-3.7250	-0.100, 0.344
Left superior parietal gyrus	-14, -64,49	45	-3.2701	0.051, 0.630
Right gyrus rectus	3, 21, -23	99	-4.0634	0.031, 0.767
Left medial frontal gyrus, orbital part	-12, 57, -1	48	-3.5220	0.209, 0.044
Right medial frontal gyrus, orbital part	3, 41, -11	37	-3.2421	0.108, 0.304

Left gyrus rectus	-1, 20, -23	29	-3.1069	0.089, 0.398
Right superior frontal gyrus, orbital part	13, 45, -21	29	-3.9485	0.136, 0.194

Note: MNI, Montreal Neurological Institute; +, positive; –, negative; Y-BOCS, the Yale-Brown Obsessive Compulsive Scale
